# Symbols in motion: Flexible cultural boundaries and the fast spread of the Neolithic in the western Mediterranean

**DOI:** 10.1371/journal.pone.0196488

**Published:** 2018-05-01

**Authors:** Solange Rigaud, Claire Manen, Iñigo García-Martínez de Lagrán

**Affiliations:** 1 CNRS, UMR 5199 –PACEA, Université de Bordeaux, Bâtiment, Allée Geoffroy Saint Hilaire, Pessac, France; 2 CNRS, UMR 5608 –TRACES, Université Toulouse–Jean Jaurès, Maison de la Recherche, 5, allées Antonio-Machado, Toulouse Cedex 9, France; 3 Arcadia-Fundación General de la Universidad de Valladolid FUNGE-UVa, Valladolid, Spain; New York State Museum, UNITED STATES

## Abstract

The rapid diffusion of farming technologies in the western Mediterranean raises questions about the mechanisms that drove the development of intensive contact networks and circulation routes between incoming Neolithic communities. Using a statistical method to analyze a brand-new set of cultural and chronological data, we document the large-scale processes that led to variations between Mediterranean archaeological cultures, and micro-scale processes responsible for the transmission of cultural practices within farming communities. The analysis of two symbolic productions, pottery decorations and personal ornaments, shed light on the complex interactions developed by Early Neolithic farmers in the western Mediterranean area. Pottery decoration diversity correlates with local processes of circulation and exchange, resulting in the emergence and the persistence of stylistic and symbolic boundaries between groups, while personal ornaments reflect extensive networks and the high level of mobility of Early Neolithic farmers. The two symbolic productions express different degrees of cultural interaction that may have facilitated the successful and rapid expansion of early farming societies in the western Mediterranean.

## Introduction

The transition to farming corresponds to the process by which human groups switched from hunting and gathering wild resources to food production based on farming and stockbreeding. In the Near East, sedentism, agriculture and herding progressively took place 12,000 years (y) ago (12ky cal BP). From there, farming technologies spread across Europe from 8,800 until 6,500 y ago (8.8ky–6.5ky cal BP) [1–3]. Several models currently assume significant regional variations in the dispersal process, combining demic diffusion of various magnitudes [4–7] with cultural transmission between incoming farming populations and local foragers [8–11]. Studies also indicate that farming technologies did not spread as a unique package, but rather that material productions followed distinct evolutionary paths with different rates of innovation and diffusion dynamics [12–14]. The cultural geography of Europe appeared completely reshaped by the diffusion of the Neolithic, due to intense and repeated inter-group interactions and the circulation of individuals, ideas and techniques [15–17].

Radiocarbon dates indicate that the coastal spread of the Neolithic in the western Mediterranean of Europe took place at a much faster rate (above 5 km.y−1) than in Central Europe [18]. The role of long-distance maritime travel combined with demic expansion and interactions with local hunter-gatherers best explains the rapid spread of farming technologies in this area of Europe [18,19]. Cultural data also identify the Mediterranean area as a hotspot of cultural diversity, where early farming populations played an influential role in the shaping of the European cultural geography [20].

However, questions persist on the relations established between the successive incoming farming populations and the role their interactions had on the gradual assimilation of the local foraging communities. To tackle this issue, we developed a statistical approach to conjointly document the processes that led to variations between Mediterranean archaeological cultures and the micro-scale processes responsible for the transmission of cultural practices within archaeological cultures.

By examining two categories of material culture, namely pottery decorative techniques and personal ornaments, we explore spatial and temporal variation in Early Neolithic cultural diversity in western Mediterranean Europe (8ky–6.5ky cal BP).

Both pottery decorative techniques and personal ornaments are intended to be seen and to transmit various symbolic messages. Pottery production and design represent a valuable social medium to signal membership and individual identity and aptitudes [21–24]. Exchange networks and social relations established between individuals and communities result in the spatial patterning of the pottery design distributions [25–27]. Personal ornaments also represent a visual message intended to mediate many social conventions related to individual and group identity [28]. Personal ornaments are used for social transactions [29,30], rituals [31] and the transmission of social memories [32,33], and to display social status within communities [34].

These two categories of production have the advantage of widespread dissemination over Europe during the transition to farming, and are characterized by a wide range of morphological, typological and stylistic traits. Their preservation in the archaeological record and their extensive analysis allow us to investigate spatial patterning and diffusion processes [17,20,35–37]. Pottery decorations and personal ornaments also fulfill exclusively symbolic functions appropriate to detecting cultural affinities between populations and patterns of cultural change through time [38–40].

Pottery attributes are classically used to define the cultural affiliation of archaeological assemblages [26,41] as well as investigate social dynamics [23,24] and characterize past cultural networks [42,43]. The ability of personal ornaments to characterize the circulation networks [44,45], social organization [46–48] and cultural geography of past populations has also been demonstrated [20,35,36].

The transfer of cultural traits within and between past communities has long been the subject of investigation, and mechanisms at work in the processes of cultural transmission and diversification are studied in depth through the application of evolutionary biology models to the transmission of cultural traits [49,50]. Cultural evolution theories state that the emergence, persistence and loss of cultural traits over time are impacted by cultural selection processes, selection bias and cultural drift [39,40,51]. Analytical methods applied to cultural evolution have contributed to the documentation of variation patterns in the archaeological record and the various transmission mechanisms responsible for similarities and differences among groups in space and time [14,52–55].

Our study relies on this approach. Our sample is constituted of cultural and chronological data recorded from 52 archaeological occupations covering Italy, France and Spain, and spanning over 1,300 years (worksheets A and B in S1 Dataset, Text A and Figure A in S1 File). The chronological dataset is built on 114 radiocarbon dates including 44 new direct datings of key occupations (worksheet H in S1 Dataset). Using a typological classification system that we developed for pottery decorative techniques [56,57] and personal ornaments [20,58], we submitted the data (worksheets D and F in S1 Dataset)–formatted as cultural, chronological and geographic distance matrices–to a series of multivariate analyses.

## Materials and methods

The method we developed aims to answer several questions: (1) What mechanisms of cultural transmission generated variations in the two symbolic productions? (2) Did they operate equally in time and space and (3) at identical speed? (4) Did they create sharply structured cultures or, conversely, did they generate high intra-cultural variability?

### Pottery

Decorated pottery is widely used to construct and compare chronologies in many archaeological contexts [41]. Pottery decorations, both abstract and figurative, constitute conventionalized artistic designs used to mediate many symbolic messages within societies [59]. Decorations may have religious intent related to rituals [60,61], or may depict conventional social narratives [62] related to folklore or even everyday experiences [63]. By working exclusively on decorative techniques, we minimize bias from factors relating to the original function of the assemblages (e.g. cooking or storage) potentially conditioning the shaping techniques and treatment surfaces [64]. Pottery decorations also have the advantage of being a visual proxy used to display individual skills and abilities [21]. The transmission of skills within a community tends to maintain decorative traditions [65], but efforts from crafters to distinguish themselves from each other also initiate changes in decorative attribute diversity in the long run [66]. The visual aspect of decorative attributes may vary depending on the tools used to decorate the artifacts, the gesture used to apply the tools to the surface of the pottery and eventually the morphology of the decoration itself. Each archaeological assemblage included in the database is described with 13 quantitative variables regarding these decorative attributes (worksheet E in S1 Dataset, Figure B in S1 File).

By considering the relative proportion of decorative technique types within a stratigraphy, it is possible to observe the introduction, growing usage and demise of specific technological and decorative traits [43,57]. Considering solely qualitative data prevents the observation of diachronic and synchronic variability that may be perceptible at different geographic scales [57]. To avoid the limitations of qualitative sampling, each variable was counted from each occupation included in the database.

We analyzed decorative techniques of pottery from 39 Early Neolithic occupations in western Mediterranean Europe (worksheets A, D and E in S1 Dataset).

The pottery remains included in the database are mostly of local origin [42], but a small number were imported from 7 km to more than 100 km away [42,67]. Both local and imported potteries are discarded items left in domestic structures by Neolithic farmers, suggesting that they were used for daily activities [68,69]. Geographic coordinates, cultural affiliation and corresponding time span of existence (worksheet H in S1 Dataset, Text A and B in S1 File) are documented for each archaeological assemblage. Our sample covers seven archaeological cultures attributed to early farming communities spanning a temporal range of ca. 1,500 years (ca. 7950~6450 cal BP).

### Personal ornaments

Body ornaments are central to the creation of social and self-identity [70]. Their various associations and their display on the body contribute to negotiating identities and unifying or distinguishing communities [28,71]. Because the primary role of personal ornamentation is to be seen in order to transmit symbolic messages, the most important characteristics of beads are those whose modification will distort their visual impact [34,72–74].

The typology of personal ornaments in this study was established following the method of Rigaud et al. (2015). It takes into account cross-cultural studies on the classification of beads and criteria used to classify archaeological artifacts [75,76]. Discrete bead types were created with reference to raw material, morphology, system of suspension (e.g. perforation or groove), size, section and profile. In the case of animal teeth, we also considered anatomical and species identification (Figure C in S1 File).

The amount and types of ornaments recovered at an archaeological site depend on the site function (e.g. domestic or funeral) and the excavation methods. Systematic sieving of the sediment with small mesh grids can, for example, significantly increase the number of small beads recovered [77]. Since, due to the above reasons, proportions of ornaments recovered at archaeological sites cannot be considered representative of the importance attributed to specific ornament types by foraging and farming populations, in our analyses we only used the presence or absence of data.

Presence or absence of bead types was coded as “1” or “0” respectively, to produce a matrix of 58 archaeological occupations coded across 88 binary traits (worksheets B, F and G in S1 Dataset). Geographic coordinates, cultural affiliation and corresponding time span of existence (worksheet H in S1 Dataset and Texts A and B in S1 File) are documented for each archaeological assemblage. Our sample covers eight archaeological cultures attributed to early farming communities spanning a temporal range of ca. 1,500 years (ca. 7950~6450 cal BP).

### Unit of analysis

Each archaeological occupation is assigned to one of the six Mediterranean Early Neolithic archaeological cultures. Archaeological cultures are defined as a system of transmission of social information that materializes population-level processes [78]. They represent geographic and chronological units characterized by archaeological occupations associated to durable material culture generated by consistent transmission across generations [49,79]. Archaeological cultures cannot be compared to cultural groups as observed in the ethnographic record, but they represent the unit of analysis commonly used for diachronic archaeological studies [14,80–82]. Early Neolithic archaeological cultures considered in this analysis are defined in the literature according to lithic technology, settlement pattern, ceramic productions and level of admixture with local foraging communities (Text A in S1 File [9,83,84]).

### Cultural diversity

We first quantified how archaeological sites differed in pottery and personal ornament attributes by using two different distance indices. The Jaccard distance index [85] is appropriate for presence/absence data [86] and has been used for calculating pairwise site differences according to their bead-type diversity (worksheet L in S1 Dataset). The Bray–Curtis distance index [87] is appropriate for count data [88] and has been used for calculating pairwise site differences according to their pottery attributes (worksheet I in S1 Dataset).

### Relationships among archaeological cultures and sites

Phylogenetic methods are effective in testing hypotheses about the role of cultural transmission in shaping material culture diversity [89,90]. However, the application of phylogenetic tools directly borrowed from evolutionary biology in order to explore cultural evolution is somewhat flawed because cultural traits are transmitted through time from generation to generation (vertical transmission) but also between neighboring populations (horizontal transmission) [91,92]. The NeighborNet technique explores evolutionary relationships characterized by potentially high levels of lateral transfer that are not perceivable through other network-based methods [93]. We used the NeighborNet method [94] to visualize the relationships among the archaeological sites and cultures. The analysis determines conflicts within the data as represented by reticulations or joining among branches. From the shape of the NeighborNet network, it is possible to infer the level of borrowing and convergence that occurred between the different archaeological sites and cultures. The analysis was performed in SplitsTree4 using standard settings [95]. The Delta score and the Q-residual [96] are two methods for calculating the level of reticulation in the network [97]. The two scores range from 0 to 1 with increasing conflicting signal.

### Isolation by distance

Understanding how isolation facilitates the development of behavioral barriers to cultural flow requires incorporating chronological and geographical distances into analyses of cultural transmission and differentiation [14,20,98]. The isolation-by-geographic-distance model predicts a positive relationship between increased cultural differentiation and spatial/temporal distance. To account for the effect of spatial and temporal distances on cultural diversity, correlation between cultural, geographic and chronologic distance matrices was calculated using a partial Mantel test [99]. The partial Mantel test has been implemented in the vegan [100] and ecodist [101] packages in R (R Core Team, 2013) using 1,000 random permutations of the data. Great-circle (spatial) distance has been calculated from the latitude and longitude data (R command published by Shennan et al., 2015, worksheets J and M in S1 Dataset). We used the Euclidean distance between earliest and latest dates for each archaeological culture (worksheets K and N in S1 Dataset). As the Mantel test results could be biased by the non-linear relationships between distances, we performed analyses using both the original untransformed and log-transformed distance matrices [102–104].

### Cultural geography

The various transmission processes and differential impact of the isolation by distance on the two productions may lead to distinct geographic patterns of diffusion. Spatial interpolation is one of the GIS processing methods used for the visualization of variations in the data, data structures and static data patterns [105–107].

Pairwise cultural distance calculated between sites was entered into a Principal Coordinates Analysis (PCoA) performed in the software PAST [108] to identify and plot similarities in terms of bead type and pottery decoration diversity between sites (Figure D in S1 File). Mapping of the two first axes of the PCoA was performed by using the Inverse Distance Weighting (IDW) interpolation method [109] run through the software QGis 2.6.1. The based map was created from ETOPO1 Global Relief Model data (http://www.ngdc.noaa.gov/mgg/global/) [110].

### Cultural structure

Variation between archaeological cultures documented by the analyses conducted in this study may hide a higher and significant variation within cultures. The AMOVA framework [111] provides a means of exploring how variability in productions is structured within and between cultures [14,112,113]. The result is expressed by the ΦST statistic, which represents to what extent the two productions are characterized by distinct archaeological cultures (worksheet C in S1 Dataset). The analysis was performed on the pottery decoration and personal ornament attributes to calculate the cultural diversity within and between archaeological cultures. The ΦST statistic was calculated with a permutation test (1,000 iterations) using the pegas package in R [114].

### Inter-culture distances

The differential success of cultural innovations contributes to material culture variation [115]. Cultural choices and mechanisms similar to natural selection may conjointly act in the shaping of cultural diversity [40]. The pace of the emergence and preservation of new cultural traits may then slow down or accelerate cultural evolution. Possible selection process was investigated by verifying if the two sets of cultural data changed at different rates [40,116]. The central tendency (mean and median) of the ornament and pottery inter-culture distance matrices was measured and two conservative nonparametric tests were undertaken: a Wilcoxon signed-rank test for paired cultures using the MASS package in R [117] and a Sign test for paired cultures using the BSDA package in R [118]. To compare the rate of evolution of the two material productions, the two tests were performed on a subset based on the seven cultures common to both datasets.

## Results

### Relationships among cultures and archaeological sites

**Fig 1:**
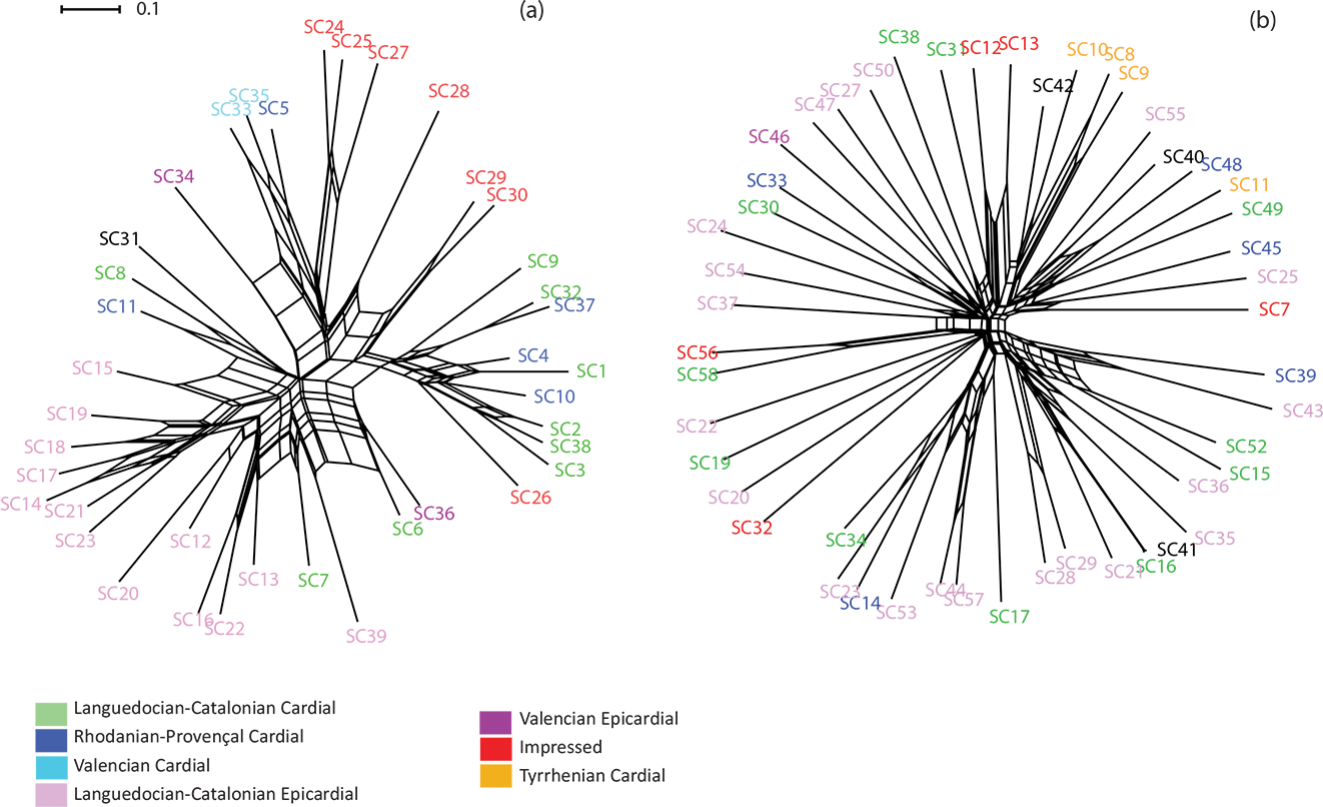
NeighborNet tree of the Early Neolithic archaeological sites identified in the western Mediterranean region: a) pottery data, b) personal ornament data. Archaeological sites are color-coded according to their cultural attribution.

The NeighborNet analysis (Fig 1) processed with the archaeological sites as the unit of analysis demonstrates that ornament and pottery data do not appear to be tree-like and display reticulations. The two networks show differences in delta score and q-residual (delta score for the ornament network: 0.30, q-residual: 0.007; delta score for the pottery network: 0.33, q-residual: 0.021). The delta score indicates a conflicting signal in the two productions with a moderately tree-like tendency for the two overall patterns. The q-residual, and to a lesser extent the delta score, suggest that reticulation is more common in the pottery data than in the ornament data. Those values are agree with previous similar studies on linguistic phylogeny [96,119,120]. The NeighborNet graph obtained for pottery data (Fig 1A) does not show clear splits, but the archaeological occupations are to some extent organized according to their chrono-cultural attributions. The most recent western occupations (in pink) are grouped on the left of the graph, and the earliest eastern occupations (in red) on the right. The other archaeological occupations belong to several other intermediate archaeological cultures and randomly cluster between the two extremes sets of occupations. The NeighborNet graph obtained for the ornament data (Fig 1B) does not show clear geographic or chronological clustering.

### Correlation in space and time

Correlations between the cultural and the spatial and temporal distances show different results for the ornament and pottery data (Table 1). Variation in the personal ornaments shows a statistically significant correlation with the geographic matrices (p < 0.01), with approximately 15 to 25% of the variance explained by geography. The temporal distance between archaeological sites does not significantly contribute to the variance. On the other hand, pottery data show significant correlation for distance in space and time: approximately 23 to 28% of the variance is explained by the geography (p < 0.01) and 27 to 37% of the variance is explained by the chronology (p < 0.01). Log-transformed distances showed similar results for both datasets.

These values are clearly higher than those observed in similar analyses of genetic distances between individuals across Europe [121,122], folktale and linguistic distances [112] and stylistic distances between other sets of archaeological data [14].

**Table 1:** Results of the partial Mantel test.

	*Pottery*		*Ornaments*	
*Variable*	*R2*	*p*	*R2*	*p*
**Geography, holding time**	0.238	0.003	0.151	0.009
**Geography, holding log(time)**	0.302	0.001	0.161	0.008
**Log(geography), holding time**	0.254	0.001	0.25	0.001
**Log(geography), holding log(time)**	0.284	0.001	0.259	0.001
**Time, holding geography**	0.336	0.001	0.021	0.35
**Time, holding log(geography)**	0.373	0.001	-0.007	0.562
**Log(time), holding geography**	0.277	0.001	0.018	0.321
**Log(time), holding log(geography)**	0.293	0.001	-0.043	0.88

### Cultural geography

**Fig 2:**
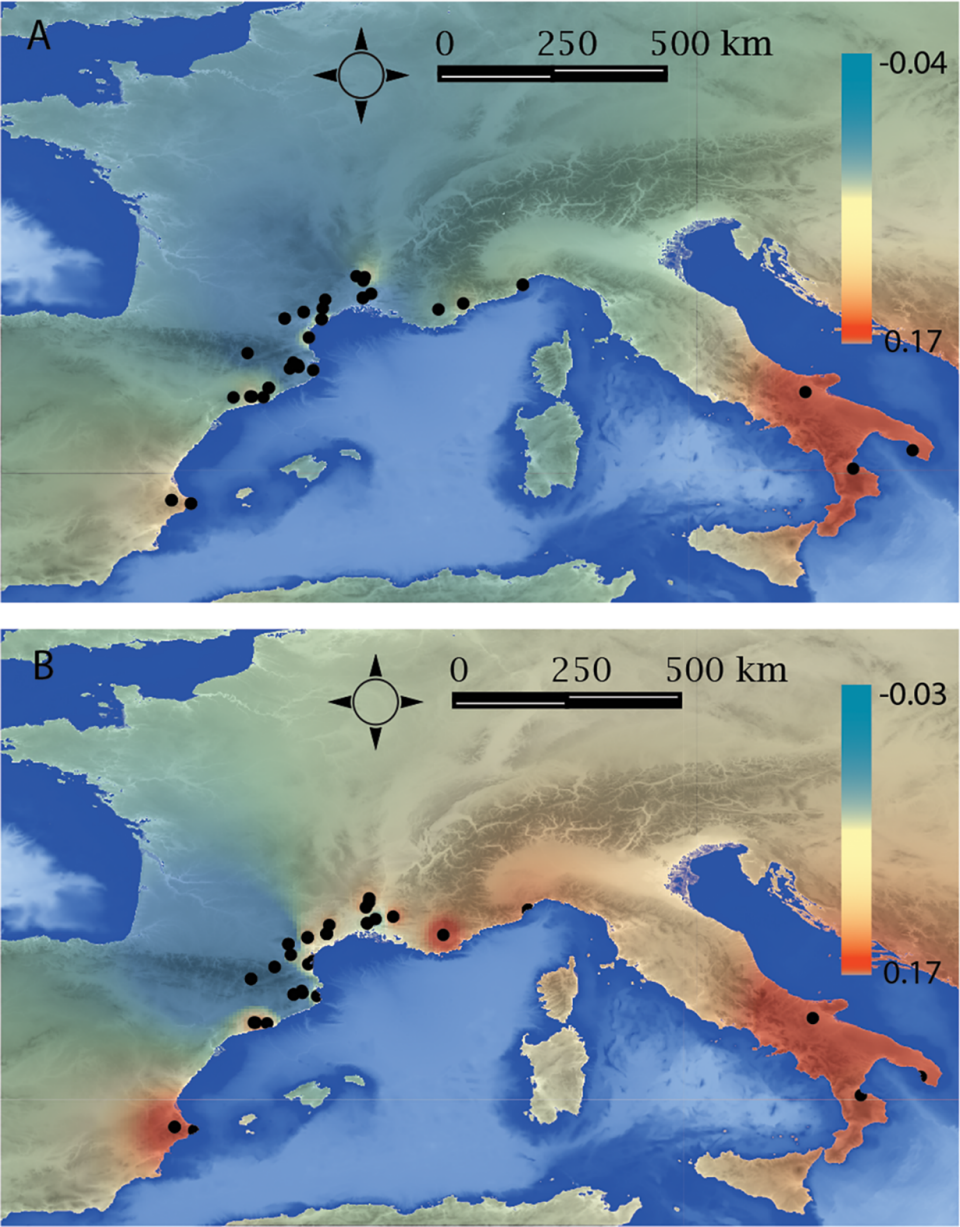
Mean Inverse Distance Weighting (IDW) interpolation of the first two axes of the Principal Coordinates Analysis (Figure D in S1 File). Diversity of the pottery attributes (A) and bead-type associations (B) express two different cultural geographies. Maps were made by S. R. using the software QGIS 2.6.1 and Etopo1 Digital Elevation Model **[110]**.

The maps of interpolated pottery decorative techniques and bead-type diversities throughout the western Mediterranean show the highest interpolated values in southern Italy (Fig 2B). Hotspots restricted to the east of the Rhône Valley in southern France and eastern Iberia are also visible on the map of bead-type association diversity. Conversely, southern France and eastern Iberia are characterized by lower interpolated values on the map of pottery decorative techniques diversity (Fig 2A).

### Cultural structure

The ΦST statistic indicates that both ornaments and pottery show a statistically significant cultural structure, but pottery shows a higher score: ΦST = 0.236 for pottery, and ΦST = 0.05 for the ornament data, both with p < 0.001. The higher score obtained for pottery data indicates that pottery decorative techniques are more variable between archaeological cultures than bead-type associations.

### Inter-culture distances

**Fig 3:**
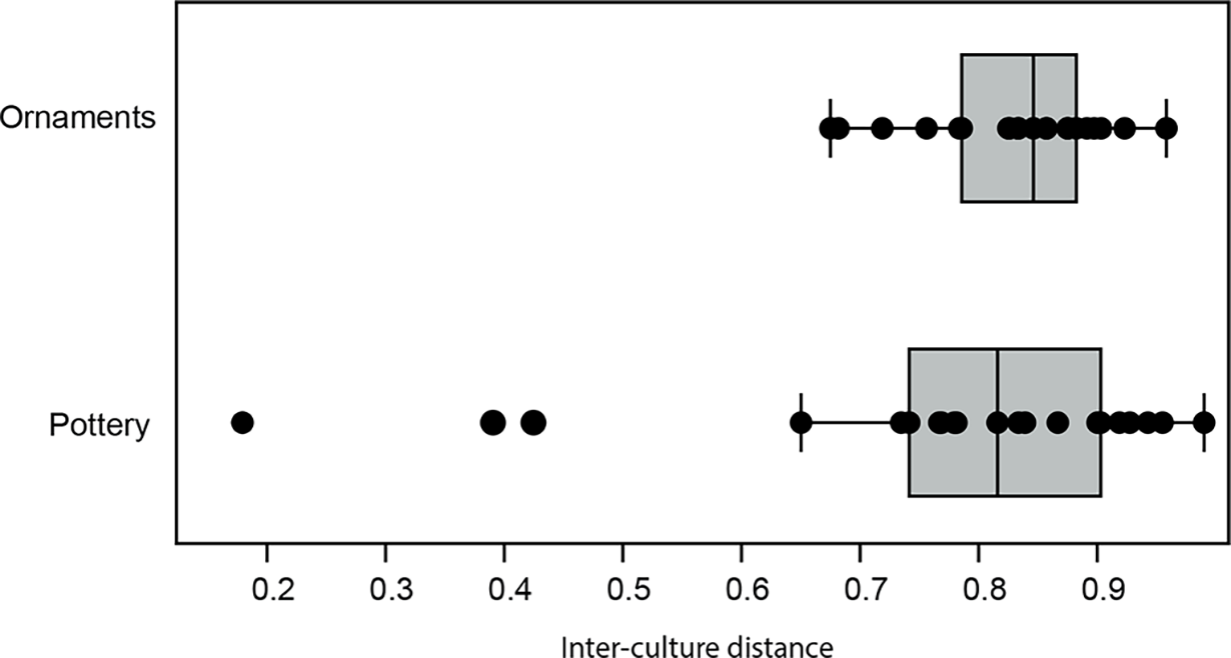
Box plot of the inter-culture distances for the ornament and pottery data.

The two tests do not indicate statistically significant differences between the paired culture distances observed in the pottery data compared to those seen in the ornament data (Wilcoxon signed-rank test: V = 97, p-value = 0.539; Sign test: s = 9, p-value = 0.663). The measures of central tendency for the ornament data (mean = 0.83; median = 0.84) are very close to those observed for the pottery ones (mean = 0.76; median = 0.81, Fig 3).

## Discussion

The NeighborNet analysis demonstrates that the cultural data do not appear to be tree-like. The reticulations show conflicting signals that might be caused by hybridization, coalescence and noise in the data [123]. In the case of cultural data, even if noise in the data cannot be excluded, the lateral flow of cultural traits is generally proposed to explain conflicting signals [112,124,125]. Lateral transmissions likely also acted in the past and played a role in the shaping of pottery decorative techniques and personal ornament diversity within early farming cultures. The different delta scores, q-residuals and clusters obtained for pottery and ornament attributes indicate separated and non-linear evolutionary trajectories for the two categories of material cultures. Studies on modern populations have shown that cultural traits and productions with a similar range of delta scores (between 0.29 and 0.41) were inclined to circulate and borrow, with increasing levels of conservatism toward the lower scores [96,125,126]. The higher delta score, and even higher q-residual, obtained for the pottery NeighborNet analysis indicates that pottery attributes were more inclined to circulate and be exchanged than personal ornaments.

The higher coefficient correlation obtained between pottery and geographic distance indicates that decorative attributes related to pottery decoration tools and techniques tend to be more distinct with increasing geographical distance between archaeological sites than bead-type associations. The impact of spatial distance on the pottery attributes indicates that the circulation and borrowing of cultural traits predominantly occurred at local level. Conversely, the capacity of ornaments to be diffused over large distances is confirmed by the lower correlation coefficient calculated between geographic distance and bead-type associations. The Mantel correlation indicates that less than 40% of the variance of the two productions is explained by geographic distance. These correlations imply that isolation by distance (IBD) affected the pottery decorative techniques and personal ornament diversity conjointly with other evolutionary mechanisms. Few other attempts have been made to test the effect of IBD on early archaeological productions [14,20,37]. Many scenarios have to be tested to better understand the impact of geographic distance on archaeological assemblage dissimilarity, including mobility (foraging versus sedentary societies), the effect of latitude and environment and the type of productions (functional or symbolic).

Distance in time also significantly predicts pottery attribute similarity, but does not contribute to an explanation of personal ornament similarity. The distance in time between archaeological sites is calculated from direct absolute radiocarbon dates (worksheet H in S1 Dataset), implying that the influence of time in the diversity of pottery decorative techniques does not simply reflect the use of potteries in the identification of archaeological cultures. Ethnographic studies indicate that pottery decorations especially fluctuate over generations and territories because they are often used by crafters to signal and differentiate their individual skills relatively to other group members [21,65,127,128]. Similar behavior may have existed within Mediterranean Eearly farming communities. The mastery of pottery manufacture and decoration also requires complex learning processes that contribute to the perpetuation of traditions over time [65,127]. The influential role of distance in time and space in pottery decoration corresponds to frequent cultural transmissions among individuals geographically close to each other. These small-scale transmissions have resulted in enduring stylistic frontiers between groups, largely visible in the archaeological record [37].

The absence of correlation between ornament attributes and temporal distance and the lower delta score and q-residual show the substantial stability and persistence of the bead-type associations, indicating more conservative traditions. The endurance of bead-type associations suggests that specific transmission processes favored the reliable reproducibility through time of symbolic codes expressed by personal ornaments. The exact transmission of the techniques and symbols involved in the crafting of personal ornamentations suggests that few specialized crafters were in charge of bead manufacture within early farming communities. Preservation of bead traditions also required the long-term maintenance of circulation networks for exogenous raw material supply [20,129].

The maps of interpolated pottery decoration and bead-type diversities show different cultural geographies related to the distinct cultural trajectories followed by the two symbolic productions.

As attested by the AMOVA analysis results, pottery attributes are more variable than bead-type associations between archaeological cultures. This result echoes the classic use of pottery shape and decoration to define the cultural affiliation of archaeological assemblages [26,41,130]. Bead-type association diversity is extremely low between the archaeological cultures. This result indicates that bead-type association diversity does not follow the chrono-cultural seriation of the Early Neolithic as defined by archaeologists according to other material and economic proxies (Text A in S1 File). Discrepancies between personal ornament assemblages and classic chrono-cultural frameworks has already been evidenced at other scales and for other archaeological contexts [20,35,36,131,132]. Traditionally, personal ornaments can have many different functions (e.g. amulets or markers of gender, social/biological age, wealth or social status) within communities [133,134]. The sharing of common symbolic messages, communicated by position and association of bead types on the body, contributes toward increasing the feeling of cohesion and membership [28] and constitutes a powerful social medium within communities [135,136]. The high level of variation of bead-type associations observed within Mediterranean early farming archaeological cultures indicates that personal ornaments were used to express a wide range of symbolic messages and fulfilled more diversified functions than pottery decorative techniques. The extreme variability of the bead-type associations likely reflects multiple individualistic styles probably related to the various social statuses that may have existed within the first farming communities.

Thus, while bead-type diversity attests to the intense circulation of ideas and individuals, the space and time diversity of pottery decorative techniques should be seen as evidence of group resilience to such permeability.

The Wilcoxon signed-rank and Sign tests do not show significant differences between inter-cultural distances calculated for pottery decoration and personal ornament attributes. Transmission mechanisms identified by our analyses did not differentially impact the cultural rate of change in one production relative to the other. Pottery decorative techniques and bead-type associations selected for symbolization were likely not directly adaptive. They were rather dedicated to generating and strengthening personal and group identities [137,138], and the fixation or loss of cultural traits related to their productions were affected by similar selection regimes.

## Conclusions

Our results shed light on the cultural mechanisms responsible for the complex cultural geography of the western Mediterranean during the transition to farming. Pottery decorations participated in restrained networks in which geographical proximity and local processes of transmission played an influential role. Bead-type associations were used to tell multiple stories about social identities, were especially resistant to change and are characterized by a greater stability through time and space. The high level of cultural connection between the early farming communities favored movement, interaction and exploration and likely represented a successful strategy for their rapid expansion in the western Mediterranean. Cultural boundaries persisted despite a flow of individuals and symbolic transfer across them.

Genetic studies indicate that the last foragers and the first farmers developed social and cultural relationships more closely tied than previously indicated through components of the material culture [139]. Biological data and chronological models support a pattern of diffusion implying geographically discontinuous contacts between local foragers and incoming farmers, but repeated in time [9,140,141]. This process of diffusion conjointly occurred with changes in material culture, including pottery decorations and personal ornaments. Pottery production represents a technological innovation mostly associated with the Neolithic way of life in the western Mediterranean. Pottery decorations were likely particularly sensitive to interactions, leading to their high variability in time and space in order to reinforce group membership. Conversely, personal ornaments were less inclined to change in space and time. Their production by both local foragers and incoming farmers implies different cultural readjustments that led to a completely different pattern of variation in time and space. The preservation of the foragers’ personal ornament styles (and likely also meanings) within emerging farming communities [20,58] has probably contributed to the maintenance of their stability through time and space.

The two symbolic productions appear as a polythetic set of cultural behaviors dedicated to mediating early farmer identities in many ways, and personal ornaments likely reflected the most entrenched and lasting facets of farmers’ ethnicity.

## Supporting information

S1 Dataset Database of the archaeological sites, layers, variables and radiocarbon dates used in the analysis. (XLSX)

S1 File Method and criteria used for the design of the bead-type, pottery decoration and radiocarbon date databases. (PDF)
