# Supplementary material for: Symbols in motion: Flexible cultural boundaries and the fast spread of the Neolithic in the western Mediterranean
Source: PLoS One. 2018 May 1;13(5):e0196488. doi: 10.1371/journal.pone.0196488 (PMC5929525; doi:10.1371/journal.pone.0196488)
Supplement: S1 File — (PDF) [file pone.0196488.s002.pdf]

Symbols in motion: Flexible cultural boundaries and the fast spread of the Neolithic in the western Mediterranean

S. Rigaud,[i] C. Manen[ii] and I. García-Martínez de Lagrán[iii]

[i] CNRS, UMR 5199 – PACEA, Université de Bordeaux, Bâtiment B8, Allée Geoffroy Saint Hilaire, 33615 Pessac, France, srigaud17@gmail.com.
[ii] CNRS, UMR 5608 – TRACES, Université Toulouse – Jean Jaurès, Maison de la Recherche, 5, allées Antonio-Machado, 31058 Toulouse Cedex 9, France, claire.manen@univ-tlse2.fr.
[iii] Arcadia-Fundación General de la Universidad de Valladolid FUNGE-UVa, Pza. Sta. Cruz 5, 47002 Valladolid, Spain, igmtzl@gmail.com.

SUPPLEMENTARY TEXTS AND FIGURES

SUPPLEMENTARY TEXT A: Genesis and development of farming economies in the western Mediterranean 1
SUPPLEMENTARY TEXT B: Datasets 2
SUPPLEMENTARY FIGURES 3
SUPPLEMENTARY FILES 6

SUPPLEMENTARY TEXT A: Genesis and development of farming economies in the western Mediterranean

Chronological and archaeological data indicate that the Early Neolithic in the Mediterranean first manifested itself in a fast and patchy pioneer colonization by culturally cohesive communities of farmers, spreading rapidly by maritime coastal travel (1–3). A slower gradual process of diffusion, likely related to a demographic expansion, followed the pioneer colonization of the western Mediterranean region and inland areas. This secondary process progressively induced the rise of technical, economic and social diversity in the farming communities all along the Mediterranean region (4, 5).

The spread of the Neolithic from southern Greece stopped between 8400 and 8000 cal BP before reaching the area of the Ionian and Adriatic seas (6). It then spread patchily across the Italian peninsula to reach the northwest of Italy and the south of France around 7700 cal BP (1). The impressed decoration of ceramics is the common denominator of the first farmers of the western Mediterranean (7). This type of decoration requires a large diversity of tools (e.g. shell, stick, bone, comb, flint) and techniques to draw various motifs. The very rich impressed pottery decorations contrast with the smooth and monochrome Early Neolithic ware of western Greece.

In southern Italy, the settlements of the first farmers are characterized by large enclosure pits, with communities relying on agriculture and herding (8). In Liguria, Neolithic occupations emerged preferentially in zones neglected by local foraging communities, probably as a consequence of the poor quality of local siliceous raw materials (1). Between 7900 and 7600 cal BP, interactions with local foraging communities, if they occurred, are not visible in the archaeological record. In southern France, the earliest Neolithic manifestations (around 7800–7600 cal BP) are due to small groups of settlers of Italic origin. Very few occupations attributed to these earliest phases of the Neolithic have been excavated so far, and archaeological remains attest to settlements of limited duration. The highly

heterogeneous economies and pottery and weaponry productions are likely the result of cultural influences from the Ligurian Neolithic, the southern Italian Neolithic and the Tyrrhenian islands (2). Significant changes in pottery production and decoration associated with more diversified economies and settlements characterize the Tyrrhenian Cardial between 7700 and 7200 cal BP (4). Sites are mostly located along the Mediterranean shores of Tuscany and Liguria and on the Tyrrhenian islands (Radi, 2010).

The Cardial formation in the Franco-Iberian area, around 7400 to 7200 cal BP, is a secondary process probably driven by the influence of the Impressed groups and the Tyrrhenian Cardial group. In few hinterland areas, Cardial is contemporary with the local Mesolithic populations, implying potential interaction with local foragers gradually converted to the economy of production. Pottery decorations consist of various types of impressions made mainly with a shell, organized in ribbons and filled with geometric motifs such as crosses and chevrons (9). The Cardial culture in southern France and Spain is characterized by a variety of economic systems and a highly mobile system of resource exploitation (10–12). It is well documented in coastal territories, but early continental penetration along main fluvial corridors is also attested. Variations in pottery production and decoration and siliceous raw material management has led to the identification of several smaller Cardial cultural units, including the Rhodanian-Provençal Cardial (1), the Languedocian-Catalonian Cardial (9) and the Valencian Cardial (13).

The latest culture of the Early Neolithic, the Epicardial, is considered an emanation of the Cardial in France (Manen and Guilaine, 2010). Epicardial pottery is characterized by decorations made of grooves and impressions arranged in bands, bundles or garlands. Epicardial culture gradually expands into all the areas previously settled by farming communities as well as beyond the limited Cardial distribution. The Epicardial is identified in the Alps, the Causses region of France and continental Iberia, including the Meseta and the large valleys toward the Portugal. Variation in the pottery decorations and settlement diversity identify two smaller French-Iberian Epicardial units: the Languedocian-Catalonian Epicardial and the Valencian Epicardial (9, 14, 15). The Balma Margineda site located in Andorra is an isolated case. The site is dated to 7500–7400 cal BP, and no artifact clearly belonging to one of the Early Neolithic archaeological cultures has been recovered during the excavations between 1979 and 1991 (16). This site is however of primary importance and attests to the earliest farming intrusions in the western Mediterranean.

SUPPLEMENTARY TEXT B: Datasets

Our recent works conducted on the western Mediterranean, including new fieldwork, archaeological collection revisions, stratigraphic controls and resampling for new direct dating, provide an exceptional opportunity to work on high-resolution and almost exclusively first-hand data.

Data recorded on the pottery decorations have been primarily directly acquired by two of us (9, 17). Personal ornament data were also directly acquired on three sites (Supplementary File S2) or alternatively mostly collected from recent publications (18–20). One archaeological site included in the dataset was excavated by one of us (21), who also actively participated in the excavation of four other sites from the

dataset (22). Other collections were recently reassessed and the stratigraphy controlled, and for some of them new direct dates were collected.

The chronological dataset used in the analysis is built on 116 radiocarbon dates, including 54 new direct datings of key occupations (Supplementary S8) and several published syntheses dealing with the time frame of the Early Neolithic expansion into the western Mediterranean (23, 24). Forty-height occupations recorded in the pottery and ornament datasets were directly dated. Radiocarbon dates obtained from 118 short-life samples extracted from material attributed to clear stratigraphic contexts were preferentially selected (24). Isolated dates were calibrated using a standard deviation of 95%. Occupations for which several radiocarbon dates were available were dated according to the sum probability distribution of the calibrated dates. Radiocarbon dates were calibrated using the software OxCal 4.3 and the IntCal13 calibration curve (25). The 15 occupations for which radiocarbon dates have been obtained from inappropriate material or for which no radiocarbon date is available were indirectly dated according to material production cultural attributes.

SUPPLEMENTARY FIGURES

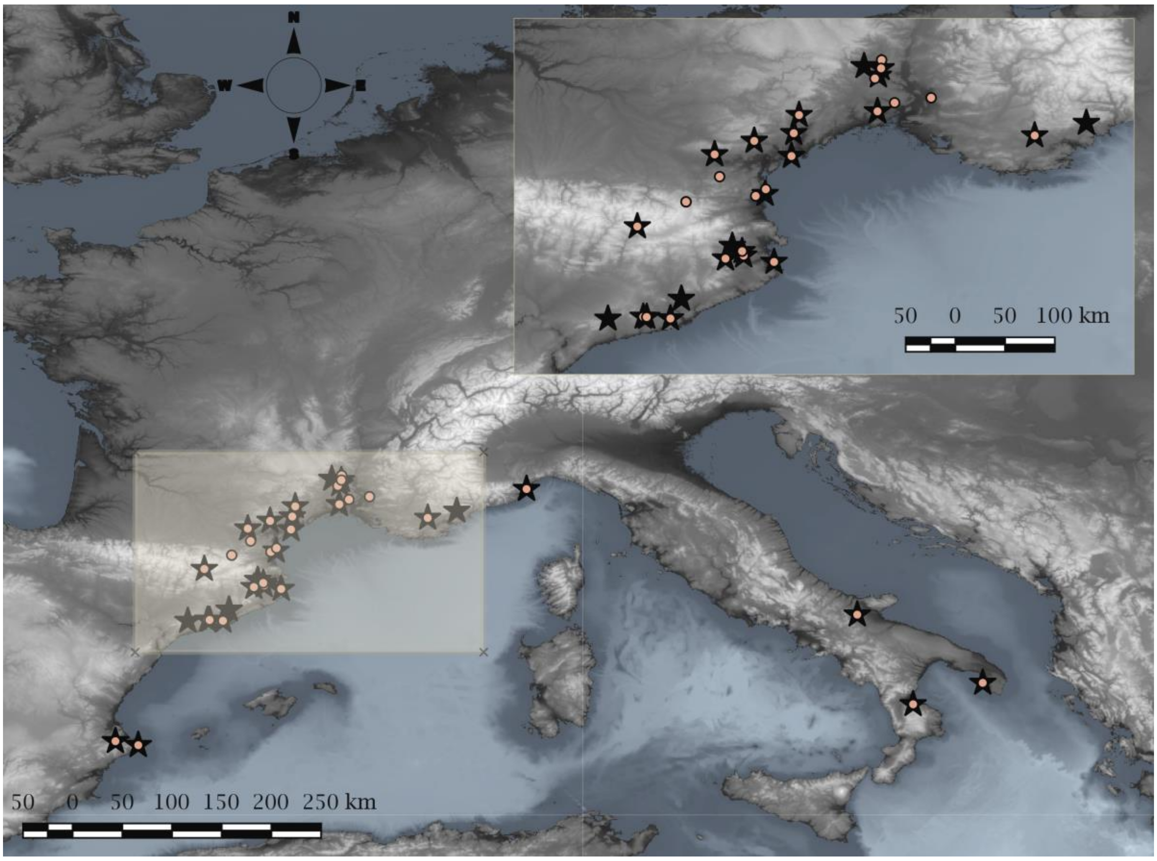

Supplementary Figure A: Location of the Early Neolithic sites recorded in the database. Black stars: pottery sites; orange dots: ornament sites. . Maps were made by S. R. using the software QGIS 2.6.1 and Etopo1 Digital Elevation Model **(26)**.

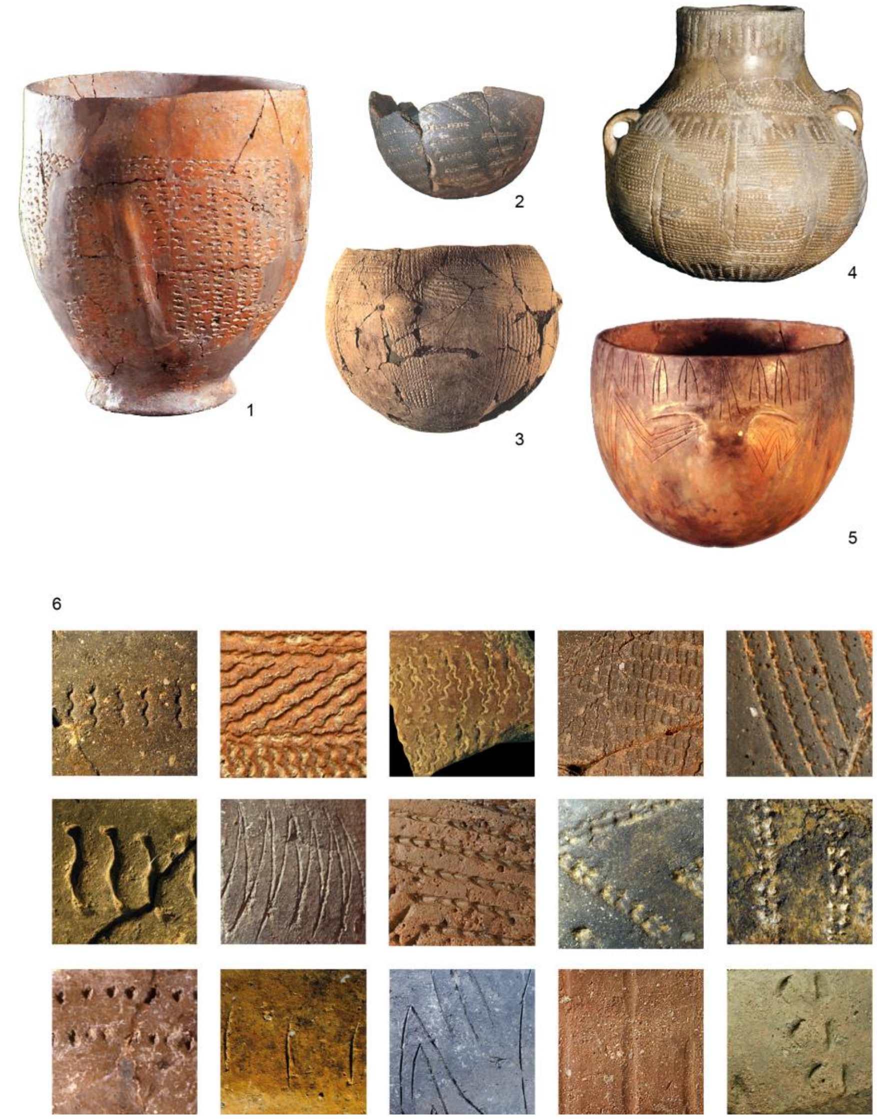

Supplementary Figure B: Western Mediterranean Early Neolithic pottery. (1) Ripabianca di Monterado (Italy, Impressed Ware, **(8)**); (2) Pont de Roque-Haute (France, Impressed Ware, **(27)**); (3) Fontbrégoua (France, Rhodanian-Provençal Cardial, **(28)**); (4) Cova de l'Or (Spain, Valencian Cardial, **(29)**); (5) Gazel (France, Languedocian-Catalonian Epicardial , **(30)**); (6) detail of the diversity of the motifs and designs identified in Early Neolithic pottery.

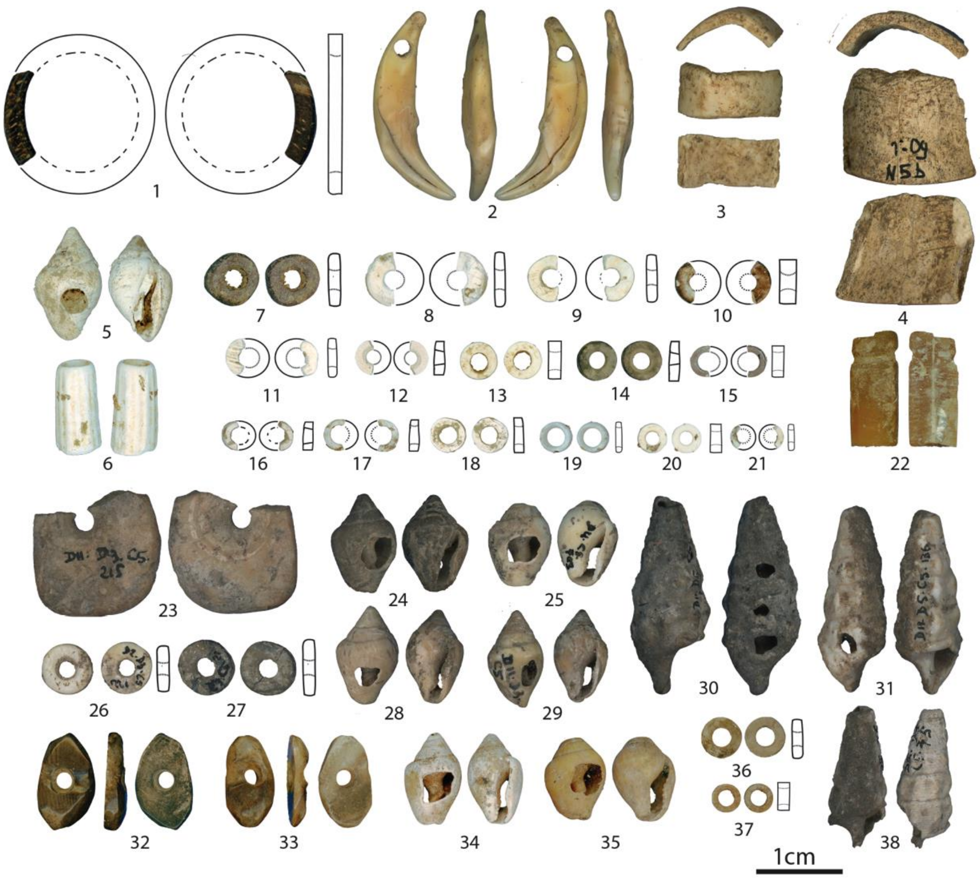

Supplementary Figure B : Western Mediterranean Early Neolithic personal ornaments: (1–22) Le Taï, France, Languedocian-Catalonian Epicardial; (23–31, 38) Roc de Dourgnes, Frane, Languedocian-Catalonian Epicardial; (32–37) Balma Margineda, Andorra. (1) stone ring; (2) fox canine; (3, 4) bone ring; (5, 24, 25, 28, 29, 34, 35) *Columbella rustica*; (6) *Dentalium* sp.; (7, 26, 27) discoid stone beads; (8-21, 36, 37) discoid shell beads; (23) stone pendant; (30, 21, 38) *Cerithium* sp.; (32, 33) oval stone beads.

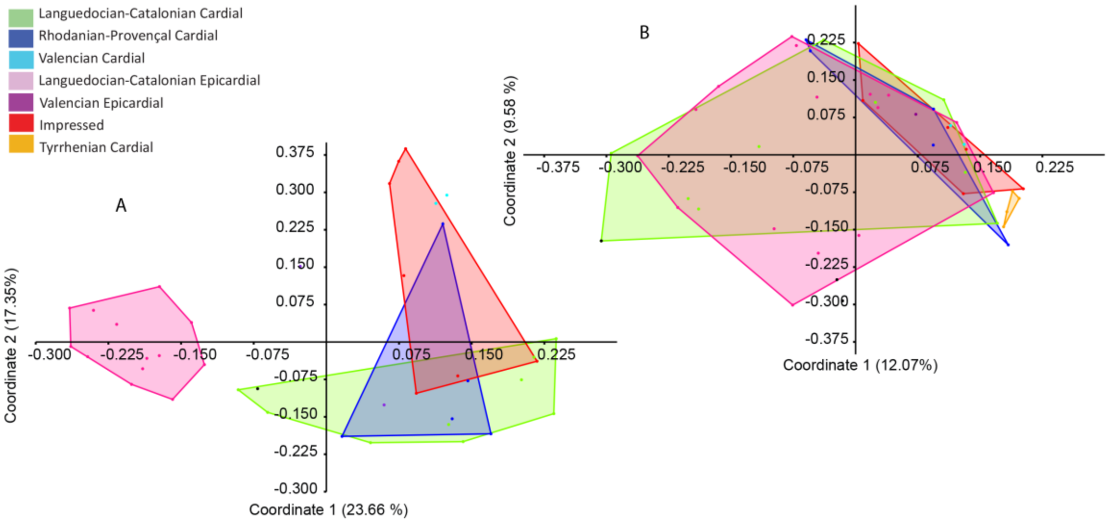

Supplementary Figure C: Principal Coordinate Analysis (PCoA) of the pairwise cultural distance calculated between sites using pottery decoration attributes (A) and bead-type associations (B).

SUPPLEMENTARY FILES

S1: Pottery sites
S2: Ornament sites
S3: Archaeological culture codes
S4: Pottery matrix
S5: Pottery attribute codes
S6: Bead-type matrix
S7: Bead-type codes
S8: Radiocarbon date database

References1. Binder D, Maggi R (2001) Le Néolithique ancien de l'arc liguro-provençal. *Bull Société Préhistorique Fr* 98(3):411–422.

2. Guilaine J, Manen C (2007) From Mesolithic to early Neolithic in the western Mediterranean. *Going over: The Mesolithic-NeolithicTtransition in the North-West Europe*, eds Whittle A, Cummings V (Proceedings of the British Academy, London), pp 21–51.

3. Manen C, Convertini F (2012) Neolithization of the Western Mediterranean: Pottery productions, circulation and recombination. *Actes : Xarxes Al Neolític : Congrés Internacional : Xarxes Al Neolític. Circulació i Intercanvi de Matèries, Productes i Idees a La Mediterránia Occidental (VII-III Mil.Lenni AC)*, Rubricatum ; 5., eds Borrell M, Borrell Tena F, Bosch Argilagós J, Clop Garcia X, Molist Montaña M (Museu de Gavà, Gavà), pp 363–368.

4. Manen C, Perrin T (2009) Réflexions sur la genèse du Cardial "Franco-ibérique." *De Méditerranée et d'ailleurs... Mélanges Offerts à Jean Guilaine* (Archives d'Ecologie Préhistorique, Toulouse), pp 427–443.

5. Rojo Guerra MA, Garrido Pena R, Garcia-Martinez-de-Lagran I (2012) *El Neolítico en la Península Ibérica y su contexto europeo* (Madrid). Editorial Cátedra.

6. Guilaine J (2003) *De la vague à la tombe. La conquête néolithique de la Méditerranée* (Seuil, Paris).

7. Guilaine J (2007) Du concept de céramique imprimée méditerranéenne à la genèse du Cardial. *Pont de Roque-Haute : Nouveaux Regards Sur La Néolithisation de La France Méditerranéenne*, eds Guilaine J, Manen C, Vigne J-D (Archives d'Ecologie préhistorique, Toulouse), pp 21–38.

8. Radi G (2010) Les séquences de la céramique imprimée en Italie. *Prem Sociétés Paysannes Méditerranée Occident Struct Prod Céramiques*:133–147.

9. Manen C (2002) Structure et identité des styles céramiques du Néolithique ancien entre Rhône et Èbre. *Gall Préhistoire* 44:121–165.

10. Bouby L (2004) Reply to : Archaeobotanical Evidence for the Spread of Farming in the Eastern Mediterranean, by Sue Colledge, James Conolly, and Stephen Shennan. *Curr Anthropol* 45:48–49.

11. Vigne JD (2007) Exploitation des animaux et néolithisation en Méditerranée nord-occidentale. *Pont de Roque-Haute (Portiragnes, Hérault). Nouveaux Regards Sur La Néolithisation de La France Méditerranéenne*, eds Guilaine J, Manen C, Vigne J-D (Centre d'Anthropologie (Archives d'Ecologie Pr´ehistorique), Toulouse), pp 221–301.

12. Vigne JD (2003) L'exploitation des animaux à Torre Sabea. Nouvelles analyses sur les débuts de l' élevage en Méditerranée centrale et occidentale. *Torre Sabea, Un Établissement Du Néolithique Ancien En Salento*, eds Guilaine J, Cremonesi G (Ecole Française de Rome, Roma), pp 325–359.

13. Bernabeu Aubán J, Molina Balaguer L, García Borja P (2010) Le Néolithique ancien valencien. Évolution et caractérisation des productions céramiques. *Premières Sociétés Paysannes de Méditerranée Occidentale. Structures Des Productions Céramiques.*, Mémoire de la Société Préhistorique française., eds Manen C, Convertini F, Binder D, Sénépart I (Société Préhistorique Française, Paris), pp 215–225.

14. van Willigen S (2004) Aspects culturels de la néolithisation en Méditerranée occidentale : le Cardial et l'Épicardial. *Bull Société Préhistorique Fr* 101(3):463–495.

15. van Willigen S, Hajdas I, Bonani G (2009) New Radiocarbon Dates for the Early Neolithic of the Western Mediterranean. *Radiocarbon* 51(2):831–838.

16. Guilaine J, Martzluff M (1995) *Les excavacions a la Balma de la Margineda (1979-1991) : volum III* (Minister d'Afers socials i Cultura, Andorra).

17. Manen C (2007) La production céramique de Pont de Roque-Haute: synthèse et comparaisons. *Pont de Roque-Haute(Portiragnes, Hérault). Nouveaux Regards Sur La Néolithisation de La France Méditerranéenne*, eds Guilaine J, Manen C, Vigne J-D (Toulouse), pp 151–166. Archives d'Écologie Préhistorique.

18. Bonnardin S (2009) La parure. *La Grotte Du Gardon (Ain). Volume I. Le Site et La Séquence Néolithique Des Couches 60 à 47*, ed Voruz JL (Toulouse). EHESS.

19. Bonnardin S, Perrin T, Manen C, Sejalon P (2014) Les éléments de parure du Néolithique ancien nîmois. *Le Néolithique Ancien de La Plaine de Nîmes (Gard, France)* (coed. AEP/INRAP, Toulouse), pp 311–330.

20. Poveda MO (2012) Los adornos producto de intercambio de ideas, materias y tecnología. Contactos e influencias entre las comunidades prehistóricas en el noreste peninsular entre el VI-IV milenos ane. *Congrés Internacional Xarxes Al Neolític – Neolithic Networks* (Revista del Museu de Gavà, Gavà), pp 257–264. Rubricatum.

21. Manen C, et al. (2004) Nouvelles données sur le Néolithique ancien gardois: résultats des campagnes de fouille 2001-2002 de la grotte du Taï (Remoulins). *Auvergne et Midi : 5èmes Rencontres Méridionales de Préhistoire Récente*, ed Dartevelle H (Clermont-Ferrand), pp 321–336. DRAC Auvergne, service régional de l'archéologie.

22. Briois François, Manen C (2009) L'habitat Néolithique ancien de Peiro Signado à Portiragnes (Hérault). *Journées de La SPF. De La Maison Au Village Dans Le Néolithique Du Sud de La France et Du Nord-Ouest Méditerranéen, 2003, Marseille, France*, pp 31–37. Mémoire de la Société préhistorique française.

23. Manen C, Guilaine J (2010) Aspects géographiques et chronoculturels du Néolithique ancien languedocien. *Premières Sociétés Paysannes de Méditerranée Occidentale. Structures Des Productions Céramiques. Séance de La Société Préhistorique Française (Toulouse 2007)*, eds Manen C, Convertini F, Binder D, Senepart I, pp 179–189. Mémoire dela Société préhistoriquefrançaise.

24. Manen C, Sabatier P (2003) Chronique radiocarbone de la néolithisation en Méditerranée nord-occidentale. *Bull Société Préhistorique Fr* 100(3):479–504.

25. Reimer P, et al. (2013) IntCal13 and Marine13 Radiocarbon Age Calibration Curves 0–50,000 Years cal BP. *Radiocarbon* 55(4):1869–1887.

26. Amante C, Eakins BW (2009) *ETOPO1 1 arc-minute global relief model: procedures, data sources and analysis* (US Department of Commerce, National Oceanic and Atmospheric Administration, National Environmental Satellite, Data, and Information Service, National Geophysical Data Center, Marine Geology and Geophysics Division).

27. Guilaine J, Manen C, Vigne J-D (2007) *Pont de Roque-Haute : nouveaux regards sur la néolithisation de la France méditerranéenne* (Archives d'Ecologie Préhistorique, Toulouse).

28. Demoule J-P (1990) *La France de la Préhistoire* (Nathan, Paris).

29. Marti Oliver B (1995) *Museo de Prehistoria "Domingo Fletcher Valls"* (Diputacion de Valencia, Valencia).

30. Guilaine J (1965) La grotte Gazel à Sallèles Cabardès (Aude). Note préliminaire sur les niveaux du Néolithique ancien (fouilles 1964-1965). *Bull Préhistoire Spéléogie Ariègeoises*:129–140.
